# Natural and artificial binders of polyriboadenylic acid and their effect on RNA structure

**DOI:** 10.3762/bjnano.6.138

**Published:** 2015-06-17

**Authors:** Giovanni N Roviello, Domenica Musumeci, Valentina Roviello, Marina Pirtskhalava, Alexander Egoyan, Merab Mirtskhulava

**Affiliations:** 1 Istituto di Biostrutture e Bioimmagini - CNR, via Mezzocannone 16, 80134 Napoli, Italy; 2 Dipartimento di Scienze Chimiche, Università di Napoli “Federico II”, 80126 Napoli, Italy; 3 Dipartimento di Ingegneria Chimica, dei Materiali e della Produzione Industriale (DICMaPI), Università di Napoli “Federico II”, 80125 Napoli, Italy; 4 Geomedi University, 3 Krtsanisi Street, 0114 Tbilisi, Georgia

**Keywords:** nucleopeptides, poly(rA) binders, RNA, self-structures

## Abstract

The employment of molecular tools with nucleic acid binding ability to specifically control crucial cellular functions represents an important scientific area at the border between biochemistry and pharmaceutical chemistry. In this review we describe several molecular systems of natural or artificial origin, which are able to bind polyriboadenylic acid (poly(rA)) both in its single-stranded or structured forms. Due to the fundamental role played by the poly(rA) tail in the maturation and stability of mRNA, as well as in the initiation of the translation process, compounds able to bind this RNA tract, influencing the mRNA fate, are of special interest for developing innovative biomedical strategies mainly in the field of anticancer therapy.

## Review

### Polyadenylation in RNA processing

**Figure 1:**
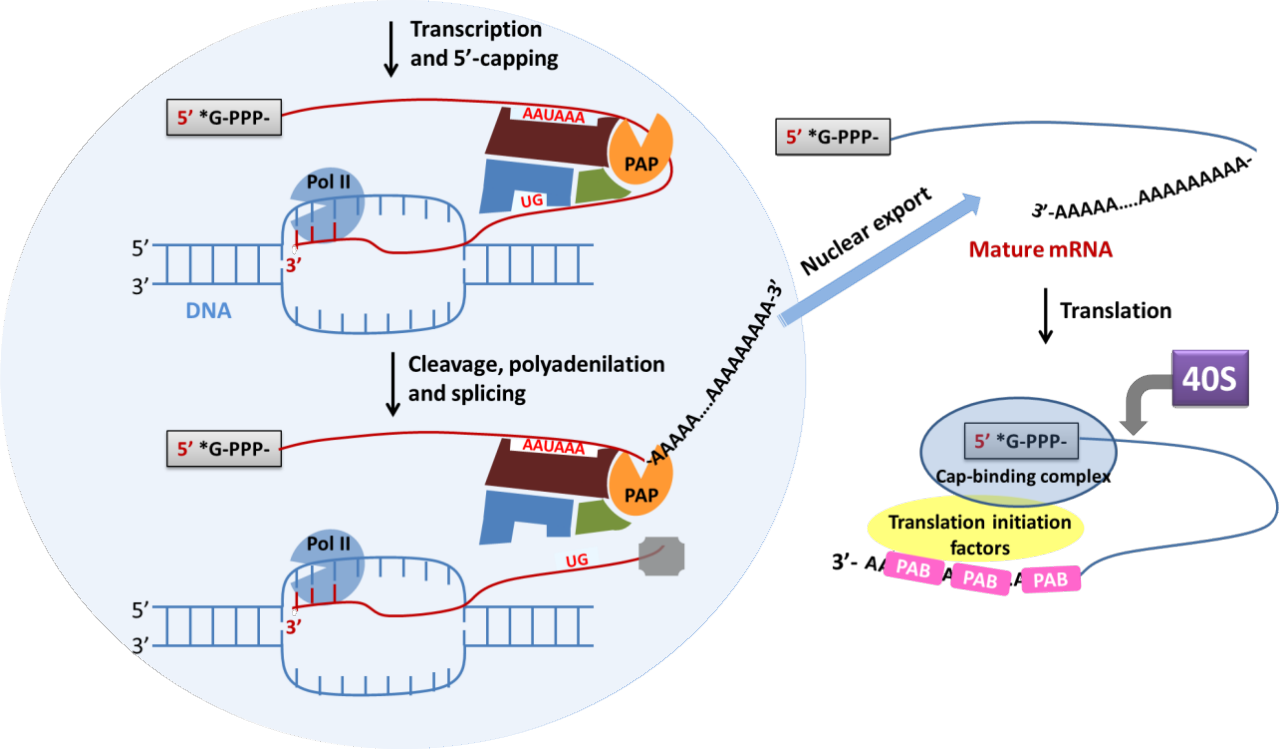
Schematic representation of transcription and translation–initiation processes in eukaryotic cells. The poly(rA) tail, added to the 3'-end of the pre-mRNA during transcription in the nucleus of eukaryotic cells, acts as the binding site in the cytosol for the polyA binding protein (PAB) which promotes translation. This recruits other proteins such as the translation initiation factor, 4G, which in turn engages the 40S ribosomal subunit.

**Figure 2:**
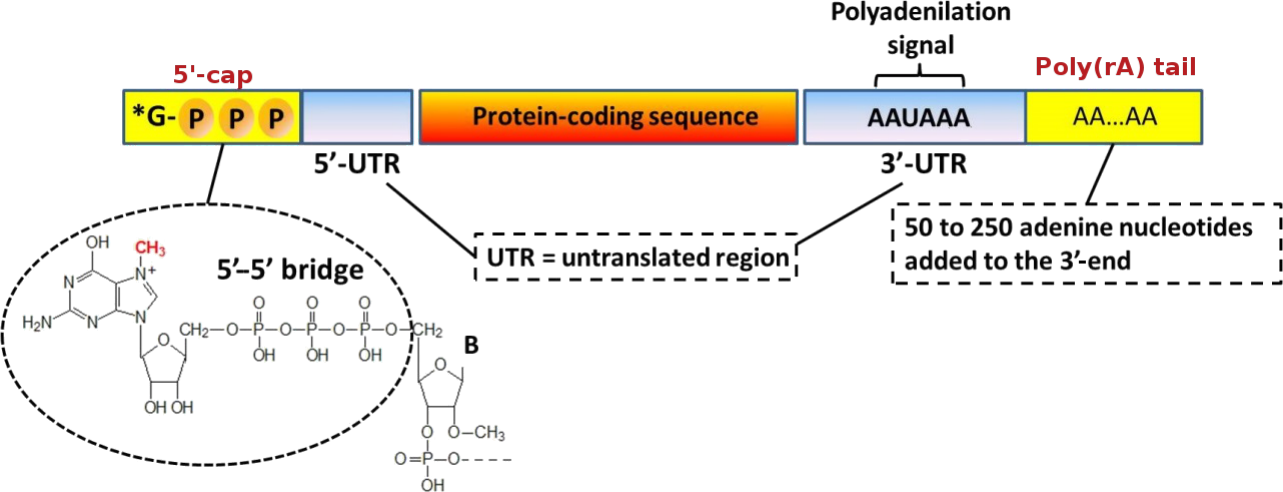
RNA processing begins with the addition of a 5'-cap and a 3'-poly(rA) tail to the pre-mRNA. The protein coding region contains coding sequences called exons. The exons are spliced together to form the mature mRNA.

Polyadenylation is part of the RNA processing pathway that leads to the production of mature mRNA molecules (Figure 1) [1]. The poly(rA) tail is a long chain of adenine nucleotides that is added to the 3'-end of the primary RNA transcript (pre-mRNA) during the transcription of a specific gene in eukaryotic cells. The pre-mRNA molecule undergoes three main processes (5'-capping, 3'-polyadenylation and RNA splicing) occurring in the cell nucleus before the RNA is exported to the cytoplasm for translation (Figure 1). The pre-mRNA undergoes capping co-transcriptionally as it is released from the RNA polymerase II exit channel. Capping is involved in several fundamental functions, such as stabilisation against exonuclease degradation, promotion of polyadenylation, splicing, transcription and nuclear export of mRNA, and it also allows the optimal mRNA translation [2–3]. The capping involves the addition of a guanosine residue methylated on the N-7 position and connected to the 5’-end of the RNA by a 5′-5′ triphosphate bridge, and the possible methylation of the 2′-OH groups of the first two nucleotides at the RNA 5′-end (Figure 2) [1].

Subsequently, transcripts are cleaved and polyadenylated at the 3′-end by means of a fine-tuned mechanism mediated by a number of RNA binding proteins and regulatory RNA motifs contained in the 3′ untranslated region (3′-UTR) (Figure 1) [4–5]. The nucleotide sequence AAUAAA typically present in the 3′-UTR, and known as “polyadenylation signal” (PAS), is recognized by the RNA cleavage complex [6]. The RNA is cleaved before the transcription termination, approximately 10 to 30 nucleotides downstream of PAS.

After the RNA cleavage is accomplished, polyadenylation starts by action of poly(rA) polymerase (PAP), which adds a chain of adenines to the RNA 3’-end [7]. Polyadenylation stops when the poly(rA) tail is approximately 100–250 bases long. This is the typical length of the poly(rA) tail [8], and the end of transcription is signaled to polymerase II [9]. Besides its role in RNA stabilization against degradation, the poly(rA) tail also allows the mature mRNA to be exported from the nucleus and translated into proteins by ribosomes in the cytoplasm. In fact, the poly(rA) tail is recognised by the poly(A) binding protein (PAB), which not only inhibits RNA degradation, but also promotes the translation of proteins in the cytosol [10]. Furthermore, PAB recruits several other factors that affect the translation process, such as the initiation factor, 4G, which in turn engages the 40S ribosomal subunit (Figure 1) [11].

Regarding the poly(rA) tail length, it is influenced by the deposition onto mRNAs of nucleophosmin [12], a multifunctional protein able to recognize G-quadruplex-forming nucleic acids [13], whose overexpression is associated with poor prognosis in ovarian cancer [14].

Since, as explained above, the poly(rA) plays a crucial role in maturation and stabilisation of RNA (as well as in the promotion of the translation process), ligands that selectively bind the poly(rA) tail could affect the function of cellular mRNA. This consequently influences the production of proteins in the cell, thus leading to new therapeutic strategies based on poly(rA) binders [15].

Interestingly, nuclear endogenous poly(rA) was tagged in living cells using fluorescently labelled oligo(dT) as a hybridization probe to study the movement of RNA through the interchromatin space [16]. Furthermore, studies performed on synthetic polyadenylic acid demonstrated that poly(rA) exists as a single strand (in random coil form) at pH 7, 0.15 M Na^+^, presenting only few stacking effects. However, it adopts a poly(rA)–poly(rA) double helix structure at pH 6, 0.15 M Na^+^ and at temperatures lower than 15 °C. In other words, variations in pH, ionic strength and temperature can provoke the structural transition of poly(rA) from the single- to the double-stranded form [17].

Nevertheless, the same conformational variation can occur also at physiological pH as a consequence of the binding of some molecular tools, as described in the present work. Interestingly, polyadenylation occurs also in prokaryotes, but in this case, it confers instability to the polyadenylated RNAs [18].

It is possible to induce self-structure in single-stranded poly(rA) by means of the binding not only of small organic molecules but also by interaction with nucleopeptides, metal–amino acid complexes, nanotubes, etc. Indeed, from a structural point of view, numerous studies were conducted in the last forty years describing in detail the properties of poly(rA) and its complexes with various molecules, such as DNA intercalators, partial intercalators and minor groove binders. The studies described in the present review allowed a better understanding of important properties, such as the RNA binding specificity, as well as the correlation between structural and thermodynamic properties. The same investigations shed light on the different binding modes between RNA and the ligands, also clarifying the influence of the ligand substituents on the formation of the RNA–drug complexes. This leads to models useful for the discovery of novel compounds suitable as modulating agents of poly(rA) structure (see the corresponding following sections for references).

### Poly(rA) as a target in anticancer approaches

As already mentioned, PAP is an enzyme with RNA polymerase activity that specifically incorporates ATP units at the 3’-terminus of mRNA. Typically, the PAP structure comprises three domains surrounding the active site, in which an ATP residue in entrance is located and subsequently used for the poly(rA) chain elongation, leading to the formation of the polyadenylic tail at the 3’-terminus of mRNA.

Another recently identified PAP, neo-PAP, is also able to modify RNA molecules with poly(rA) tails, but is specifically overexpressed in many kinds of cancer with respect to healthy cells. Moreover, neo-PAP function is regulated in a different manner with respect to the case of classical PAP. The activity of this enzyme is of fundamental importance for the mechanisms connected to the mRNA processing, as well as their potential aberrations in cancer cells [19]. Furthermore, the presence of a specific poly(rA) polymerase overexpressed in cancer cells and, more in general, the enhanced polyadenylation activity found in cancer cells, seem to indicate that poly(rA) is an important candidate for anticancer strategies [20]. Compounds that are able to bind poly(rA) could be able in principle to interfere also with the translation process, both in healthy and neoplastic cells. However, the selective inhibition of protein translation in neoplastic cells with respect to normal cells by poly(rA) binders of different nature is currently under investigation. A possibility to achieve the desired selectivity involves the conjugation of these binders with molecules (targeting agents) specific for receptors overexpressed on cancer cells in analogy to the work of Guaragna et al. concerning folate-conjugated drugs [21]. In this way the delivery of these molecular tools can be selectively directed towards cancer cells and, after their internalization, the anticancer activity could be exerted either by the entire conjugate or by the free binder after loss of the targeting agent.

Thus, due to the importance of poly(rA) binders, for example in the field of the development of novel anticancer drugs, we report a description of different classes of molecules characterized by their ability to interact with poly(rA). Some of them are of natural origin, as in the case of neomycin, palmatine, berberine, chelerythrine and other alkaloids and glucosides; others are artificial, as in the case of proflavine, 4',6-diamidino-2-phenylindole dihydrochloride (DAPI), azacyanines, nucleopeptides, benzodifurans, nanotubes and metal–amino acid complexes.

### Poly(rA) binders of natural origin and their synthetic derivatives

#### Alkaloids

**Figure 3:**
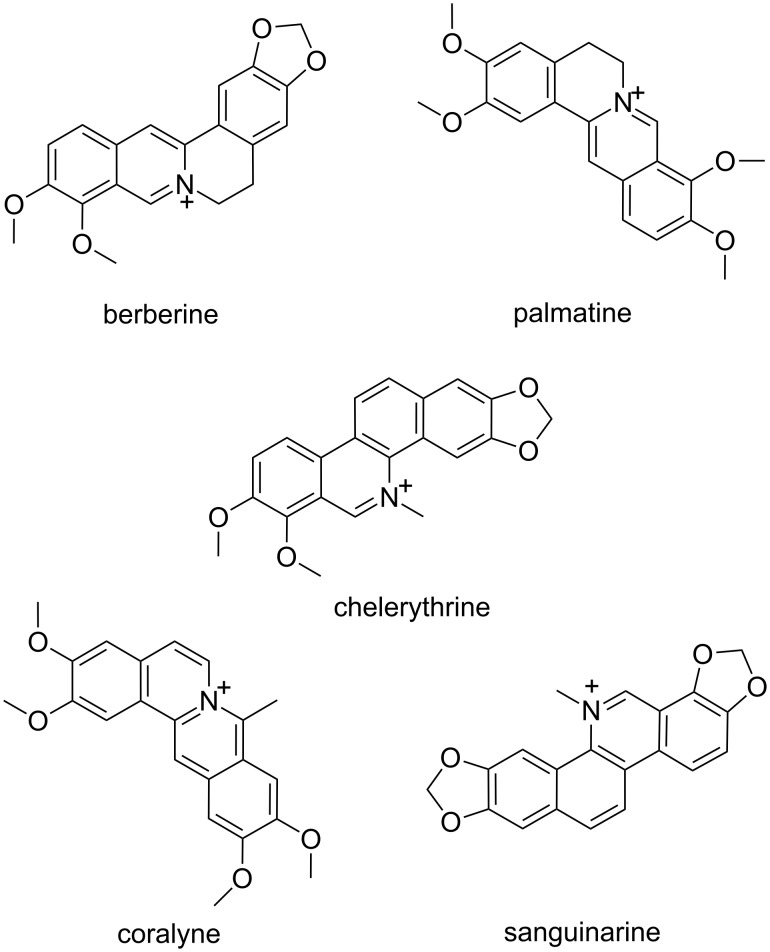
Structural representation of the alkaloids whose poly(rA) binding ability has been investigated.

**Isoquinoline alkaloids:** Alkaloids are a family of natural compounds with therapeutic potential and recently, their interaction with several kinds of RNA has been reported in the literature. For example, isoquinoline alkaloids, some of which are found in many botanical families, are of great importance in this respect. Many of these alkaloids show interesting binding abilities towards RNA structures and are currently the object of scientific investigation with the aim to develop drugs based on RNA targeting [22–23]. Among the various isoquinoline alkaloids able to bind poly(rA), particular relevance is attributed to berberine, palmatine, coralyne and sanguinarine (Figure 3). The binding process involving these molecules, whose structures bring positive charges, is characterized predominantly by electrostatic interactions with the RNA, and in some cases by intercalative binding.

The first report on the interaction of a isoquinoline compound with poly(rA) concerned berberine [24]. In particular, this alkaloid was found to bind poly(rA) with greater affinity than double-stranded B-DNA or tRNA [24]. Recently, the ability of berberine to interact with single-stranded poly(rA) was the object of another investigation presented by Yadav et al. This study took advantage of different techniques, including UV spectrophotometry, circular dichroism, spectrofluorimetry, and viscosimetry, were they were able to demonstrate a mechanism of partial intercalation for this binding [25]. The interaction process was confirmed by both hypochromic and bathochromic effects revealed in the UV–vis spectrum of the alkaloid. The binding also induced increases in fluorescence intensity and viscosity of the solutions and was accompanied by perturbations of the circular dichroism spectrum of the single-stranded poly(rA). From the spectrophotometric analysis it was shown that berberine was able to strongly bind single-stranded poly(rA) in a non-cooperative manner. On the other side, the same compound did not induce variations in the adsorption, fluorescence, and circular dichroism spectra of double-stranded poly(rA) and it did not increase the viscosity of poly(rA) duplex solutions, thus suggesting that berberine does not significantly bind poly(rA) in its double-stranded form [25].

Xing et al. [26] verified the ability of coralyne to interact with poly(rA) and also to induce self-assembled structures in this nucleic acid. Also, Giri and Kumar [27] confirmed the ability of coralyne to bind with high affinity single-stranded poly(rA), where an affinity constant of about 10^5^ M^−1^ was demonstrated by UV and fluorescence studies. Calorimetric studies additionally verified the coralline–poly(rA) binding of an exothermic nature, favoured by both negative enthalpy and positive entropy variations. The authors also reported the induction of self-assembled structures in poly(rA) due to coralyne, as evidenced by circular dichroism experiments. Remarkable changes in the circular dichroism spectrum of poly(rA) were found with complex formation of 2:1 base-pair/alkaloid stoichiometry. Çetinkol and Hud [28] studied the binding of small organic molecules with poly(rA), confirming that coralyne was able to bind with high affinity poly(rA) in a highly cooperative binding process. In fact, a minimum of six molecules were necessary to stabilize the poly(rA) structure at room temperature. This interaction produced clear transitions from random coil to structured forms of poly(rA), variable as a consequence of the coralyne concentration [28]. Thus, by simply adjusting the concentration of the ligand used in the experiments, it was possible to drive transitions in different molecular structures of the RNA.

The protoberberinic alkaloid palmatine was found to bind single-stranded poly(rA), showing an affinity constant of about 8 × 10^5^ M^−1^ [29]. Giri et al. [30] investigated the interaction of palmatine with various structures of poly(rA), both in single- and double-stranded forms, making use of different biophysical techniques. Competition dialysis, fluorescence and UV adsorption studies shed light on the molecular aspects which are at the basis of the strong and specific poly(rA)–palmatine binding. On the other hand, the interaction with double-stranded poly(rA) proved to be weaker as confirmed also by circular dichroism and viscosimetry studies. In the same study the authors demonstrated that all the protoberberinic alkaloids able to bind with high affinity single-stranded poly(rA) were characterized by a much lower affinity (10^4^ M^−1^) for double helix poly(rA) structures induced at low pH conditions.

Recent reports on poly(rA) binding activity of the plant alkaloid chelerythrine (Figure 3) indicated that this natural compound was also able to induce poly(rA) self-structures with the formation of a poly(rA) helix that showed a cooperative melting transition in circular dichroism experiments [31].

Giri and Kumar [32–33] reported that the isoquinoline alkaloid sanguinarine (Figure 3) was able to strongly interact with single-stranded poly(rA) with an association constant of about 4 × 10^6^ M^−1^. Such binding induced the formation of self-structures in poly(rA) strands and led to cooperative melting transitions, as revealed in circular dichroism, UV and calorimetry studies. Finally, the fluorescence data showed that sanguinarine acts as an intercalator, while calorimetry experiments indicated that its binding is enthalpy-driven. In another study the same authors also studied the interaction of this alkaloid, already known for its binding to DNA, with double-stranded poly(rA) [34]. From fluorescence and UV studies a strong interaction of this alkaloid with poly(rA) double helix clearly emerged, and this binding was characterized by (a) an affinity constant of about 10^4^ M^−1^, (b) a remarkable energy transfer from adenine base pairs to the ligand, as well as (c) a significant conformational variation of the poly(rA) duplex. Calorimetry studies evidenced an exothermic- and enthalpy-driven binding of sanguinarine to double helical poly(rA) in which the alkaloid acts as intercalator.

With respect to the interaction of sanguinarine with other double helical poly(rA) complexes, Chowdhury et al. [35] studied the binding of this alkaloid with different double-stranded RNAs and, among the others, with the poly(rA)·poly(rU) complex (the symbol “·” indicates Watson–Crick base pairing). UV and fluorescence studies showed that sanguinarine is able to bind the above-mentioned complex with a binding affinity of about 10^4^ M^−1^. Fluorescence quenching and hydrodynamic studies clearly indicated the intercalation activity of sanguinarine to this RNA double helix. Interestingly, the binding of sanguinarine with poly(rA)·poly(rU) occurred with an affinity higher than that observed with the analogous complexes poly(rI)·poly(rC) and poly(rC)·poly(rG), and was characterized by a significant hydrophobic contribution to the interaction process.

Regarding the interaction with mixed double helical poly(rA) complexes of the other isoquinoline alkaloids, it was shown that coralyne and berberine are able to perturb the structure of poly(rA)·poly(rU) complexes [36]. Indeed, UV and fluorescence studies revealed that such alkaloids cooperatively bind these complexes with an affinity of about 10^4^ M^−1^. Furthermore, fluorescence quenching studies evidenced that berberine and palmatine act as partial intercalators of RNA double helices, while coralyne provides a complete intercalation. The interaction with these alkaloids significantly stabilises the melting of poly(rA)·poly(rU) double helices and the binding data, obtained by melting studies, are in good agreement with those obtained by other techniques. Furthermore, among the three alkaloids, coralyne showed the highest binding affinity towards the poly(rA)·poly(rU) complex, followed by palmatine and berberine, whose affinities were almost identical [36]. Sinha and Kumar [37] conducted similar studies on the interaction of alkaloids with ternary poly(rA) complexes, investigating the binding activity of the natural berberine and palmatine, as well as of the synthetic derivative coralyne, towards a poly(rU)×poly(rA)·poly(rU) triple helix (the symbol “×” indicates Hoogsteen base pairing) through different biophysical and calorimetric techniques. In all cases, the ligands bound the triple helix in a non-cooperative manner with affinities of about 10^5^ M^−1^, in the case of berberine and palmatine, and 10^6^ M^−1^ in the case of coralyne. The alkaloids seem to stabilise the Hoogsteen bonds of the triple helix without affecting the double helix stability. Circular dichroism studies suggested a stronger perturbation of the triplex structure for the binding with coralyne with respect to berberine and palmatine [37]. Analogously, Biver et al. [38] studied the interaction of coralyne with the following poly(rA)-based complexes: poly(rA)·poly(rU), poly(rU)×poly(rA)· poly(rU) and poly(rA)·poly(rA) through the use of spectrophotometry, spectrofluorimetry, circular dichroism and viscosimetry. In this study the authors demonstrated for the first time that coralyne was able to induce a disproportion of poly(rA)·poly(rU) to poly(rU)×poly(rA)·poly(rU) triple helix or, depending on the coralyne/complex ratio, to single-stranded poly(rA). By means of circular dichroism, kinetic and melting experiments, the same authors demonstrated that this process involved first the binding of coralyne to a double helix and, subsequently, the interaction of the formed complex with another duplex furnishing a coralyne-bound triple helix and a free poly(rA) strand. With respect to the poly(rA)·poly(rA) double helix, this complex undergoes aggregation as a consequence of the interaction with coralyne but only at concentrations of double helix much higher than those of poly(rA)·poly(rU) [38].

**Modified isoquinoline alkaloids:** Synthetic modifications to the alkaloid structures can lead to molecules with interesting RNA-binding properties. For example, in the study of Islam et al. [39] the authors not only confirmed the ability of the vegetal alkaloid berberine to induce self-structure in poly(rA) strands, but also examined several alkyl ether analogues of berberine, finding that all the molecules under scrutiny were able to cooperatively bind poly(rA). More in detail, by means of circular dichroism, optical melting and dilution experiments, the same authors ascertained the formation of self-structure in poly(rA) as a consequence of the interaction with such modified substrates. Of equal importance were studies on the energy of the interactions which demonstrated that increasing the length of the alkyl chain led to binding which is more entropy-driven, and that chain length influenced the ease of self-structure formation. Recently, Basu et al. [40] found that 9-*O*-*N*-aryl/arylalkylaminocarbonylmethyl-substituted analogues of berberine showed a strong and specific binding to triple helical as well as double helical complexes of poly(rA) with poly(rU).

**Figure 4:**
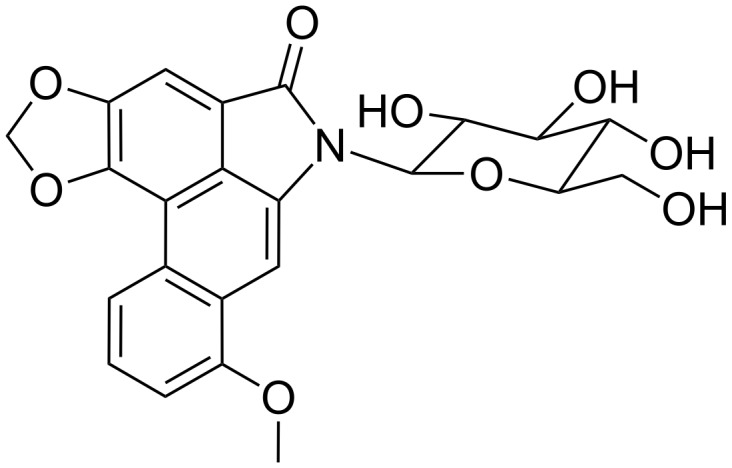
Structural representation of aristololactam-β-D-glucoside.

**Aristololactam-β-D-glucoside alkaloid:** Aristololactam-β-D-glucoside (Figure 4) is another plant alkaloid whose structure also contains a sugar moiety which is able to interact, even if only weakly, with poly(rA) without inducing any self-structure of such substrate [41].

The binding of this glucoside towards poly(rA) is non-cooperative and is characterized by an affinity of about 10^4^ M^−1^. The presence of a hydrophilic moiety, such as the sugar moiety, in the structure of this alkaloid does not seem to favour the binding with poly(rA).

#### Neomycin

**Figure 5:**
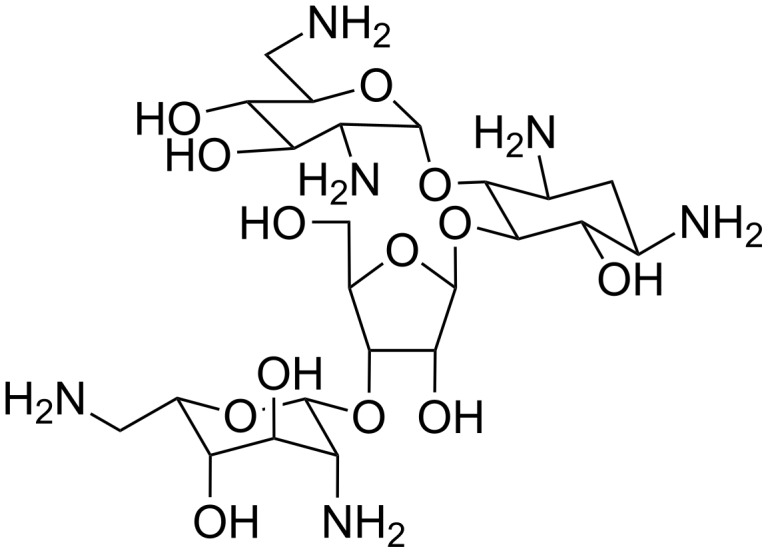
Structural representation of neomycin.

The natural aminoglucoside antibiotic neomycin (Figure 5), whose ability to interact with various structures of nucleic acids is well known, was proven to bind single-stranded poly(rA) in slightly acidic conditions with an affinity of about 10^6^ M^−1^ [42].

Melting experiments monitored by circular dichroism confirmed the formation of a complex characterized by a mp of about 47 °C. Furthermore, also double helical poly(rA) was recognized by neomycin and showed an increase in the mp of 17 °C at pH 5.5 upon binding, thus suggesting a strong stabilization of the double helix due to the interaction with the aminoglucoside. In addition, Xi et al. [43] studied the interaction of neomycin with several nucleic acid complexes by means of competition dialysis, UV, fluorescence, computational and calorimetry studies, demonstrating that this molecule is able to bind with high affinity (of about 10^7^ M^−1^) not only poly(rA)·poly(rA) but also poly(rU)·poly(rA) double helical complexes. However, a lower affinity was found in the case of the interaction of neomycin with DNA·RNA hybrid complexes such as poly(rA)·poly(dT) that showed an affinity of about 10^6^ M^−1^. The binding activity of neomycin towards nucleic acids is attributed to its cationic nature due to the amino groups protonation at pH less than 7.

#### Purine derivatives

**Figure 6:**
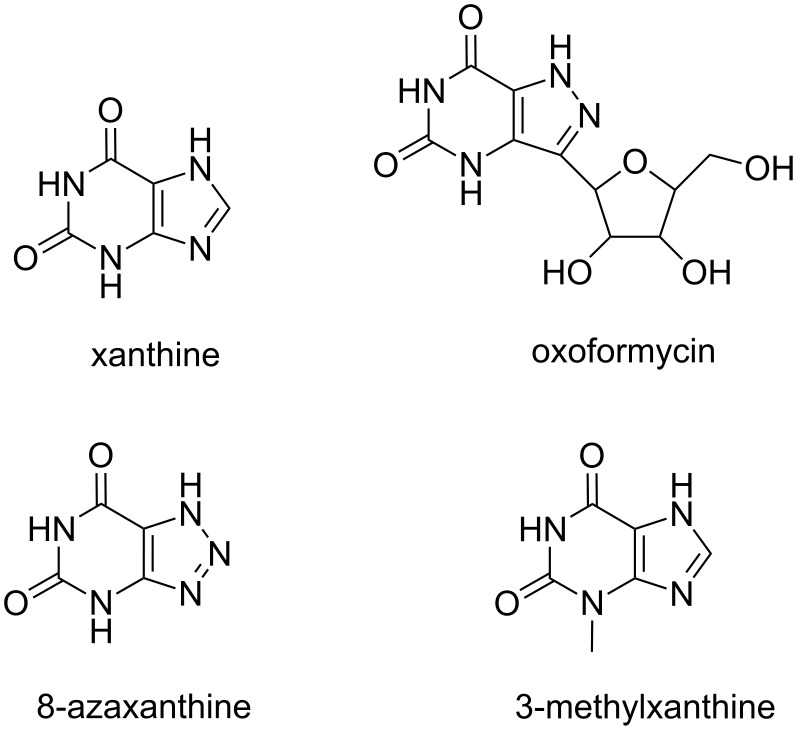
Schematic representation of the purine derivatives able to bind poly(rA), studied by Davies [17].

In the work of Davies [17] the poly(rA) recognition by different heteroaromatic compounds (Figure 6) was studied by means of several techniques, including equilibrium dialysis, optical rotatory dispersion and UV spectrophotometry. These experimental studies were conducted on neutral solutions with 0.15 M Na^+^ concentration, a condition in which poly(rA) is in the random coil form with only a low degree of nucleobase-stacking. They were also performed at pH 6 at 15 °C, a situation in which the same RNA adopts a double helical structure. At low temperature (3.5 °C) and pH 6, xanthine is able to form a 1:1 complex with poly(rA) that is different from oxoformycin that forms a 1:1 complex with the same RNA not only at pH 6 but also at pH 7.

Analogously, the same author also studied the complexes of poly(rA) with 3-methylxanthine and 7-methylxanthine finding, in this case, a different nature of the complexes formed by the two isomers with poly(rA) [17]. Interestingly, in the case of 1-methyl and 9-methyl isomers as well as 3,9-dimethylxanthine, no poly(rA) interaction was detected. The same work led also to exclude the formation of complexes of poly(rA) with hypoxanthine, allopurinol, 6,8-dihydroxypurine, theophylline and theobromine.

### Artificial poly(rA) binders

#### Synthetic organic molecules

**Figure 7:**
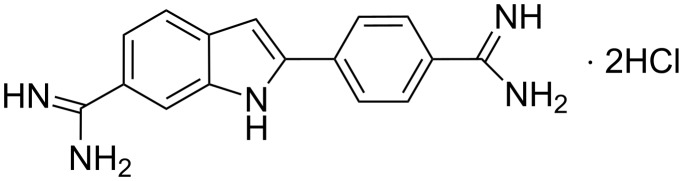
Structural representation of DAPI.

Regarding the interaction of artificial molecules with poly(rA), Manzini et al. [44] found that the compound known as 4',6-diamidino-2-phenylindole dihydrochloride (DAPI, Figure 7) can bind double helical poly(rA).

Similarly, synthetic analogues of quinoxaline are also able to bind poly(rA)·poly(rU) complexes [45], while other artificial compounds such as ethidium salts, quinacrine, and proflavine proved to be efficient binders of single-stranded poly(rA) capable of inducing self-structures of such RNA. This characteristic is absent in the compound Hoechst 33258 that acts as a poly(rA) binder, but is unable to provoke structural organization in the same RNA [46].

**Figure 8:**
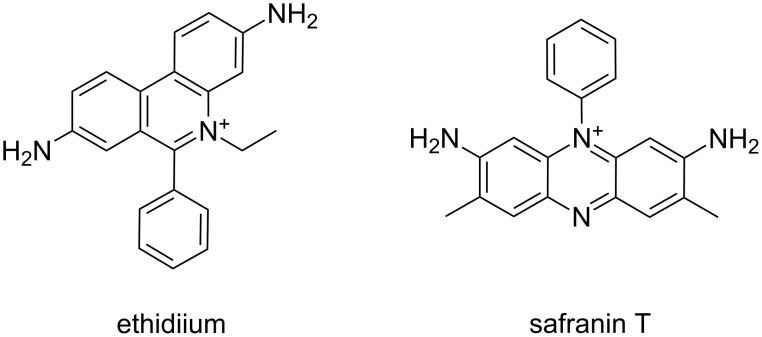
Structural representation of ethidium and safranin T cations.

Furthermore, Pradhan et al. [47] recently found that the phenazinium dye safranin T (Figure 8) recognised single-stranded poly(rA) with great affinity, while it is not able to bind the double-stranded form of the same RNA.

**Figure 9:**
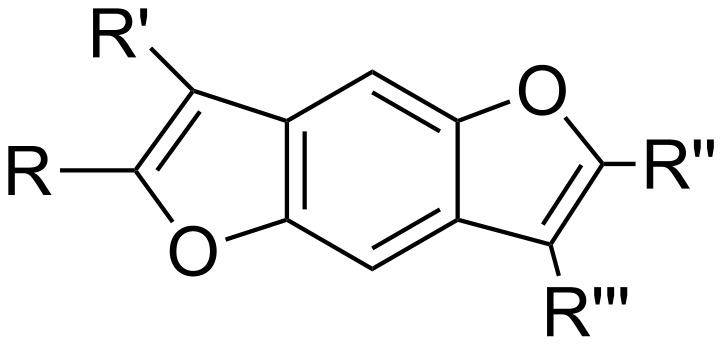
Structure of a benzodifuran core.

Interesting binding results relative to the interaction with poly(rA) of artificial compounds, such as benzodifuran (Figure 9), cationic peptides and nucleoamino acid-based molecules were found by Roviello et al. [48–56], Moggio et al. [57] and Saghyan et al. [58].

**Figure 10:**
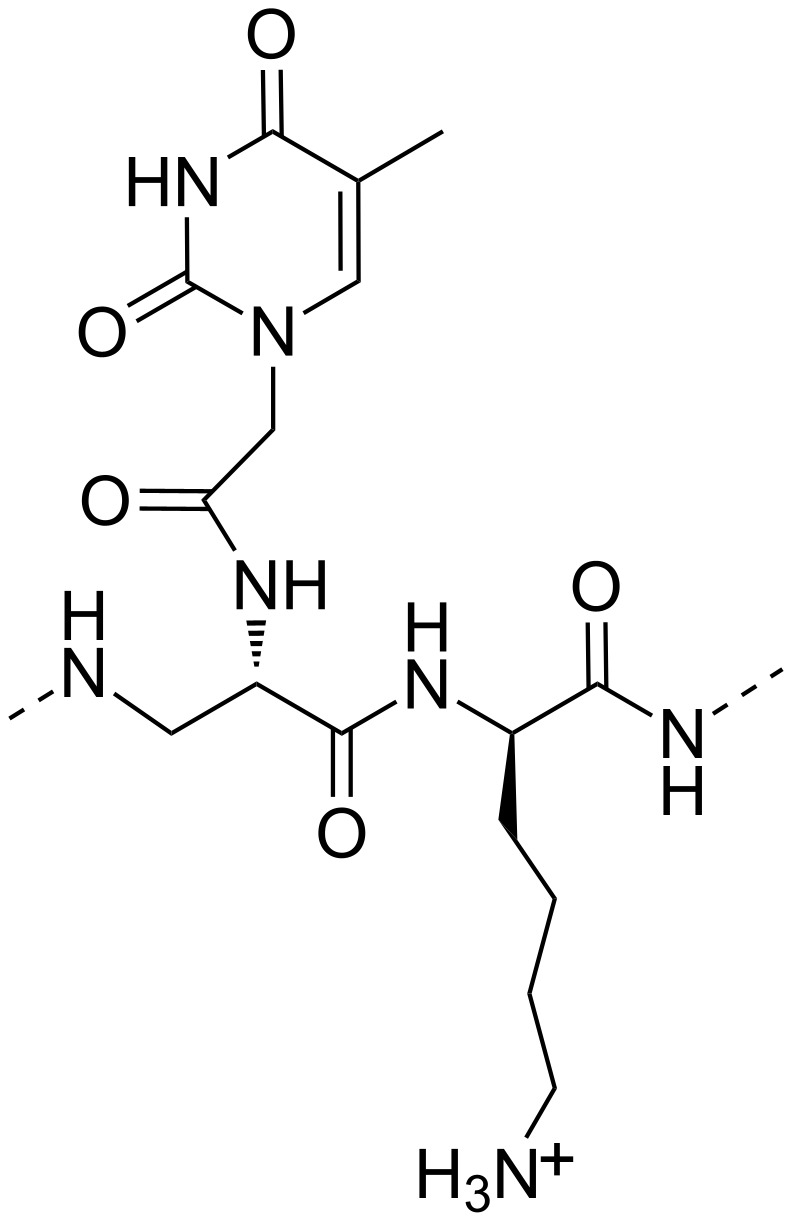
Molecular representation of a nucleopeptide able to bind poly(rA).

In particular, regarding the interaction with nucleopeptides (Figure 10), a significant role was played not only by the molecular recognition of complementary nucleobases, but also by the electrostatic interactions occurring between anionic phosphodiester moieties and the cationic residues present in the nucleopeptide structures. It is also interesting to mention that, on the other hand, negatively charged nucleopeptoids carrying a poly(dT) sequence were not able to hybridize both poly(dA) and poly(rA). This proved that electrostatic interactions associated with adequate flexibility and three-dimensional presentation of the functional groups of the backbone provide determinant contributions for efficient RNA recognition [59]. Besides the RNA binding ability, many other interesting properties of the nucleobase-containing peptide-like molecules are known in the field of biomedicine, including the antigene activity and the modulation of the expression and function of noncoding RNAs [60–61].

**Figure 11:**
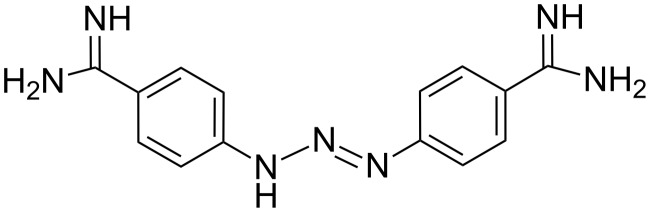
Structure of diminazene.

Diminazene (Figure 11), an antitrypanosomal drug also known by the name berenil, was found to be able to bind some DNA and RNA complexes. In particular, Pilch et al. [62] demonstrated the binding of diminazene with poly(rU)**×**poly(rA)·poly(rU) triple helices, where it acted as a ligand of the minor groove of the RNA complex without any intercalation activity.

Giri and Kumar [46] investigated the interaction of several molecules with poly(rA) to identify the structure–activity relationships for small organic DNA-binding molecules able to induce self-structure formation in poly(rA). This work concerns both classical intercalators, such as ethidium, coralyne, quinacrine, proflavine, and classical minor groove binders, such as Hoechst 33258 and DAPI. Poly(rA) binding of each of the molecules under investigation was characterized by adsorption spectroscopy titration, job plot and calorimetry, while the self-structure induction was monitored by circular dichroism, optical melting and calorimetry experiments. Giri and Kumar concluded that all the examined intercalators, except partial intercalators, were able to induce self-structure in poly(rA). Among the DNA minor groove ligands, Hoechst 33258 was able to induce poly(rA) self-structure, while DAPI proved to be ineffective. The capability of a ligand to induce self-structure in poly(rA) is related to the cooperative binding ability of the compound towards this RNA. Indeed, all molecules able to cooperatively bind poly(rA) were also able to induce self-structure in the same RNA. Finally, Gautier et al. [63] studied a new set of synthetic molecules based on an intercalating agent (i.e., oxazolopyridocarbazole (OPC)) covalently bound through a polymethylene chain of variable length to the 3'-terminus of short DNA sequences. Such compounds exhibited higher stability and specificity with poly(rA) complexes than the corresponding underivatized DNAs.

#### Nanotubes

Poly(rA) binding is not exclusive to small organic molecules. For example, Zhao et al. [64] reported an example of self-structure induction in a single-stranded poly(rA) caused by interaction with single-walled nanotubes (SWNT). More in detail, the authors demonstrated by UV, microscopy, NMR and circular dichroism studies that, upon interaction with SWNT, single-stranded poly(rA) was converted in neutral solution to its double helical form, a structure that in the absence of this poly(rA) ligand is stable only in acidic conditions*.*

#### Amino acid–metal complexes

**Figure 12:**
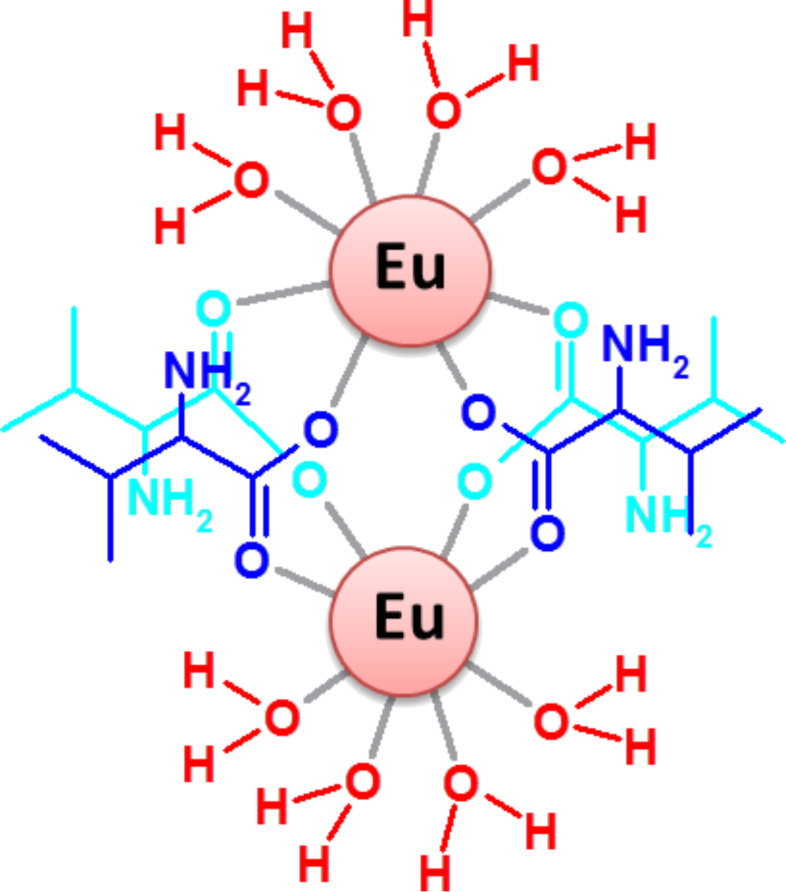
Schematic representation of a L-valine-europium complex.

Another original example of a poly(rA) binding system different from alkaloids or other small organic molecules can be found in the research work of Zhang et al. [65], where the effects on nucleic acid structures of adducts formed between amino acids and europium metal ions, was investigated for the first time. In particular, a L-valine-europium complex (Figure 12) proved to be able to bind both the DNA and RNA molecules studied by the authors.

Interestingly, this complex was able to induce self-structure in single-stranded poly(rA), and melting experiments led to the conclusion that the observed transition was cooperative similar to the case of a cooperative melting of a DNA double helix.

#### Environmental pollutants

In addition, some molecules derived from anthropic pollution are also able to bind, in some cases covalently, the polyadenylic tail present at the 3’-terminus of mRNA, an event that was linked to cancer development. Even if these molecules are clearly dangerous to human health, the study of the structural characteristics on the basis of their poly(rA) binding ability could aid in designing new drugs aimed to modulate mRNA functions. In particular, it was shown that benzopyrene-like molecules, which are formed after combustion of unsorted waste or fuels such as kerosene and naphtha, are able to covalently bind poly(rA) with affinity higher than poly(dA) DNA [66]. Also the work of Craig and Isenberg [67] evidenced similar binding results, concerning the ability of polycyclic hydrocarbons to bind double-stranded poly(rA) structures. Haloacetonitriles are chlorinated pollutants present in drinking water and swimming pools formed as disinfection by-products, whose oncogenic effect is currently under investigation [68]. Nevertheless, they are able to form adducts with poly(rA), as demonstrated in the study of Lin et al. [69] in which a covalent linkage between poly(rA) and dichloroacetonitrile was evidenced.

## Conclusion

In this review we described several molecular systems, both natural and synthetic, which are able to interact with polyadenylic acid in its single-stranded form or with its ordered forms and complexes. Among the factors which determine the ability of small organic binders to induce self-structures in poly(rA), it is worth mentioning the cooperative binding, the intercalation activity and the planarity of the molecules. For example, the non-planar nature of berberine and palmatine is believed to prevent these two alkaloids from inducing a structural organization of poly(rA) similar to that caused by the interaction with the planar coralyne. Although a great number of these binders induce self-structure in poly(rA) single strands, it is still unclear which mechanism leads to such organization. A better understanding of the mechanism is clearly desirable in view of rational design of novel poly(rA) binders. Nevertheless, targeting poly(rA) with small organic molecules may result in a very efficient route for the development of novel drug families, useful for example in anticancer therapy and/or in a variety of other innovative and powerful biomedical applications. Thus, an extensive search for potential candidates selectively targeting these peculiar RNA sequences, for instance using high-throughput affinity chromatography-based methods [70], is currently a promising strategy for the discovery of novel, more efficient and specific RNA-targeting drugs.
